# Effects of the Carbon Fiber-Carbon Microcoil Hybrid Formation on the Effectiveness of Electromagnetic Wave Shielding on Carbon Fibers-Based Fabrics

**DOI:** 10.3390/ma11122344

**Published:** 2018-11-22

**Authors:** Hyun-Ji Kim, Sung-Hoon Kim, Sangmoon Park

**Affiliations:** Department of Energy and Chemical Engineering, Silla University, Busan 46958, Korea; khyunj32@gmail.com (H.-J.K.); spark@silla.ac.kr (S.P.)

**Keywords:** absorption mechanism, carbon fiber-carbon microcoil hybridized nonwoven fabric, carbon fiber-carbon microcoil hybridized woven fabric, electromagnetic wave shielding effectiveness, nonwoven fabric, woven fabric

## Abstract

Carbon fiber-carbon microcoil (CF-CMC) hybrids were formed on carbon fiber (CF)-based fabric. The morphologies of CF-based fabrics and CF-CMC hybridized fabrics were investigated. The electrical conductivities of the CF-CMC hybridized fabrics were examined and compared with those of native CF-based fabrics. Furthermore, the electromagnetic wave shielding effectiveness (SE) of the CF-CMC hybridized fabrics was investigated across operating frequencies in the 8.0–12.0 GHz range, and the results were compared with those for native CF-based fabrics. For the CF-based nonwoven fabrics, the SE values were improved by the CF-CMC hybridization reaction, although the electrical conductivities of the nonwoven fabric were reduced by the CF-CMC hybrid formation. For the CF-based woven fabrics, the SE values were improved by more than twofold throughout the entire range of frequencies, owing to the CF-CMC hybrid formation. This dramatic improvement was partly ascribed to the enhanced electrical conductivity, particularly in the transverse direction to the individual CFs. Owing to the increased thickness of the woven or nonwoven fabrics after the CF-CMC hybrid formation and the intrinsic characteristics of CMCs, the absorption mechanism for the SE was determined for the main factor that contributed to the improvement of the SE values.

## 1. Introduction

In the era of the high frequency-required technology like fifth generation (5G) technology, mobile electronic devices often require higher operating frequencies, which are necessary for transferring enormous amounts of data using these devices. In general, absorption loss and reflection loss of electromagnetic (EM) wave radiation are regarded as the main shielding routes associated with EM interference (EMI) [1,2,3,4]. Absorption loss increases with increasing frequency, whereas reflection loss decreases with increasing frequency [3]. Therefore, for effective operation in high-frequency regimes, the shielding materials for protection against EMI should have better EM wave absorption characteristics instead of reflection characteristics.

The coil-type geometry of carbon microcoils (CMCs) has been proposed as an effective geometry for the induction of an electric current that would generate a magnetic field [5,6]. In this geometry, the electron influx of the incoming EM wave can be stopped and rotated in the generated magnetic field. In this way, the incoming EM wave can be absorbed in the CMCs which have DNA-like double helix type geometry. Thus, materials that protect against EM waves by absorbing EM waves are very much needed for use with carbon coil-based materials and/or composites [7,8,9].

For hybrid interconnections in carbon microcoil (CMC)-based hybrid materials, carbon nano coil-carbon microcoil (CNC-CMC) has been shown to contain electron-conducting channels [10]. Thus, CMC-based hybrid materials, compared with pure CMCs, can exhibit better electron conductivity, owing to their generated electron-conducting channels [11]. This improvement in the electron conductivity can improve the shielding effectiveness in a wide range of operating frequencies, covering the low to high operating frequency ranges. Uniform growth of CMCs on the surface of prepared carbon fibers was achieved by using pre-coated Ni nanoparticles on the carbon fibers in a thermal chemical vapor deposition (CVD) system [12,13]. A material with good microwave absorption was reported by using 10 wt % of the carbon microcoil–carbon fiber hybrid in a paraffin matrix [12]. From this perspective, CMC-based hybrid materials have been considered as promising materials for protection against EM wave radiation throughout a wide range of operating frequencies.

In the present work, we studied the formation and properties of carbon fiber-carbon microcoil (CF-CMC) hybrid materials on CF-based nonwoven and woven fabrics. To form the hybrid materials, Ni nanoparticles were first spread on prepared carbon fibers (CFs), following which CMCs were formed on the surfaces of the Ni-nanoparticle-covered CFs. The morphologies of the CF-CMC hybrid materials were investigated in detail. The EM wave shielding properties and the electrical conductivity values of the nonwoven and woven fabrics with CF-CMC hybrid formations were measured, estimated and discussed. Based on these results, we discuss the characteristics of the shielding effectiveness of the CF-CMC hybridized fabrics across operating frequencies in the 8.0–12.0 GHz range.

## 2. Materials and Methods

**Figure 1:**
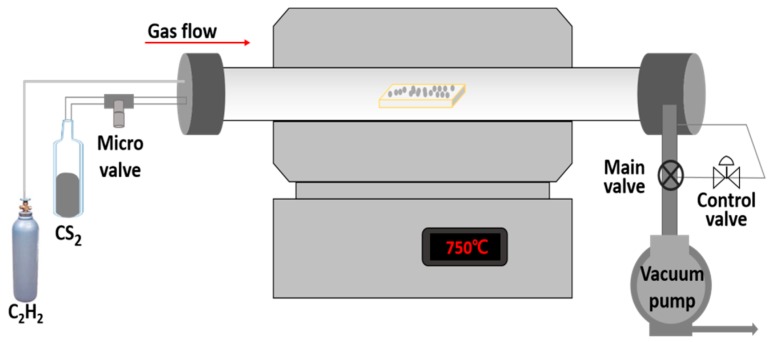
Schematic of the thermal chemical vapor deposition system equipped with the microvalve to control the injection amount of CS_2_ gas into the reactor.

As a catalyst for the formation of CF-CMC hybrid materials, approximately 0.01 g of Ni powder (99.7%), with particle diameters ranging from 0.5 to 5 μm, was spread onto the prepared CFs on a 2-mm-thick aluminum substrate. A thermal chemical vapor deposition (TCVD) system was employed for the formation of CF-CMC hybrid materials, using C_2_H_2_ as the source gas and CS_2_ as the additive gas (Figure 1). A glass tube equipped with a microvalve was designed and installed at the front part of a reactor, as shown in Figure 1. In this glass tube, liquid-state CS_2_ at room temperature was converted into gaseous CS_2_ in a vacuum. It was then accurately injected into the reactor using the precisely controlled microvalve.

**Figure 2:**
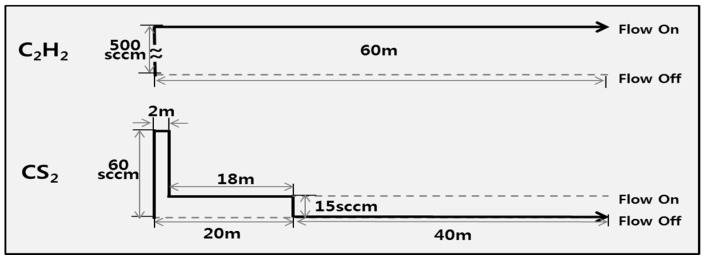
Injection process of C_2_H_2_ and CS_2_ for the formation of carbon fiber-carbon microcoil (CF-CMC) hybrid materials.

The flow rate of C_2_H_2_ was set to 500 standard cm^3^ per minute (sccm), and the flow rate of CS_2_ was set to 15 sccm. The substrate temperature and the total pressure in the reactor during the reaction were 750 °C and 100 Torr, respectively. The overall reaction time (mainly owing to the C_2_H_2_ gas) was 60 min. According to the reaction process, the CS_2_ gas was injected during the initial 20 min of the process (Figure 2). Indeed, the ratio of C_2_H_2_/CS_2_ flow was varied during the reaction process. We introduced the stepwise type injection flow scheme for CS_2_ to enhance the formation of the CMCs on the sample (see the process in Figure 2). Namely, the injection of the higher CS_2_ flow rate (60 sccm) was carried out during the initial 2 min reaction. And then, the injection of the lower CS_2_ flow rate (15 sccm) was performed during the 18 min. Finally, the injection of CS_2_ flow was stopped for 40 min. By doing this, the growth of CMCs on the fabrics could be enhanced as in the previous report [14]. The detailed reaction conditions for the formation of CF-CMC hybrid materials are listed in Table 1.

The morphologies of CF-based fabrics and their corresponding CF-CMC hybrids were investigated in detail using field-emission scanning electron microscopy (FESEM; Hitachi S-4200, Chiyoda, Japan). Crystalline phases were identified using X-ray diffraction (XRD, Shimadzu 6000, Kyoto, Japan), using CuKα radiation (λ = 0.1541 nm) in the angular range of 10° < 2θ < 70°. The quality of the carbon coils was investigated using a micro-Raman spectrophotometer (Renishaw inVia Reflex, Gloucestershire, UK) with an Ar-ion laser (wavelength, λ = 514.5 nm) in the spectral range of 1200–1800 cm^−1^.

**Figure 3:**
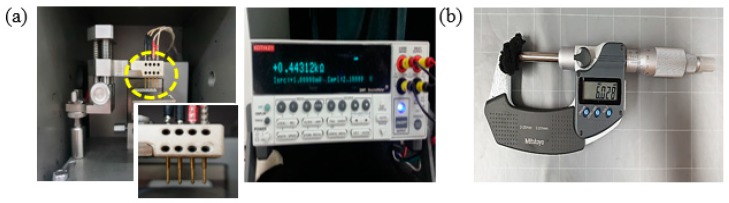
(**a**) Images of four-point probe and multimeter (**b**) Instrumental setup for measuring the thickness of samples using the micro-meter.

The resistivity values of the samples were measured using a four-point probe (labsysstc-400, Nextron) connected with a multi-meter (Keithley 2400, OH, USA), using Ohm’s law and a correction factor at room temperature (Figure 3a) [15]. The thickness of the samples was investigated using the micro-meter (Mitutoyo 406-250-30, Kawasaki, Japan) as shown in Figure 3b and verified by the cross-sectional images of FESEM.

**Figure 4:**
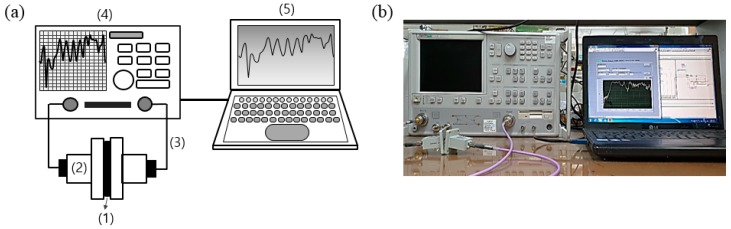
(**a**) Schematic diagram of the vector network analyzer: (1) sample; (2) wave-guide test holders (90WECAS); (3) coaxial cables (18.5 GHz, CM06A); (4) vector network analyzer (VNA); and (5) computer and (**b**) optical photograph of the experiment setup for electromagnetic interference (EMI) shielding measurement.

The shielding effectiveness (SE) of the CF-CMC hybridized fabrics was measured using a vector network analyzer (VNA, Anritsu 37369C, Kanagawa, Japan) in accordance with the wave guide method as shown in Figure 4 [7]. The measured values were compared with those of the native CF-based nonwoven and woven fabrics, and the differences are discussed below. The setup consisted of a sample holder with its exterior connected to the network analyzer (Figure 4a). A coaxial sample holder and coaxial transmission test specimen were set up according to the wave guide method. Scattering parameters (S_11_ and S_21_) were measured in the frequency ranges of 8.0–12.0 GHz.

The EMI SE was calculated from the scattering parameters by the following formulas:

SE_Tot_ = 10log (P_I_/P_o_) dB.
SE_R_ = −10log (1−R) dB
SE_A_ = −10log (T/1−R) dB

R, T, and A are the reflection, transmission and absorption coefficient, respectively. P_I_ and P_o_ are the incidents and transmitted power, respectively. SE_Tot_, SE_R_, and SE_A_ are the total, reflective, and absorptive EMI SE, respectively.

## 3. Results and Discussion

**Figure 5:**
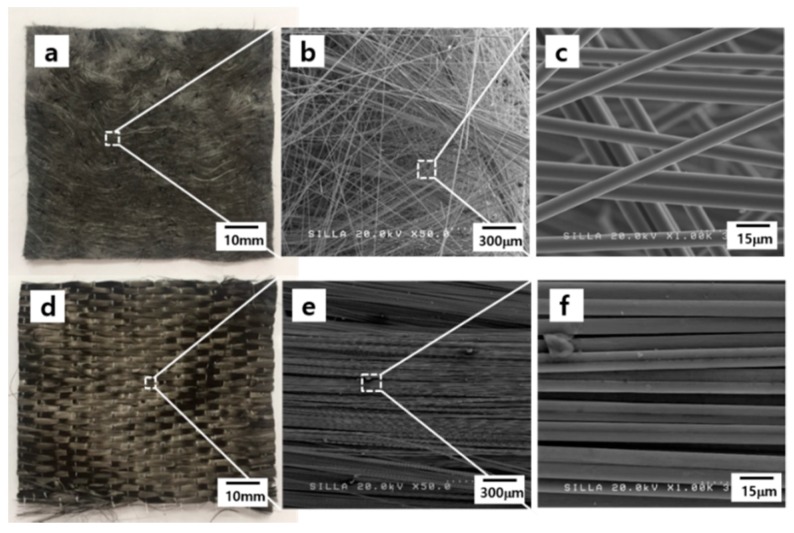
Top: (**a**) photograph; (**b**) Field-emission scanning electron microscopy (FESEM) image; and (**c**) magnified FESEM image of the nonwoven fabric. Bottom: (**d**) optical photograph; (**e**) FESEM image; and (**f**) magnified FESEM image of the woven fabric.

Figure 5 shows the photographs of CF-based nonwoven and woven fabrics, and their magnified FESEM images. The individual CFs contributing to the nonwoven and woven fabrics were ~7.5 μm in diameter. As shown in these figures, the nonwoven fabric contained randomly oriented individual CFs whereas the woven fabric exhibited well-aligned individual CFs.

**Figure 6:**
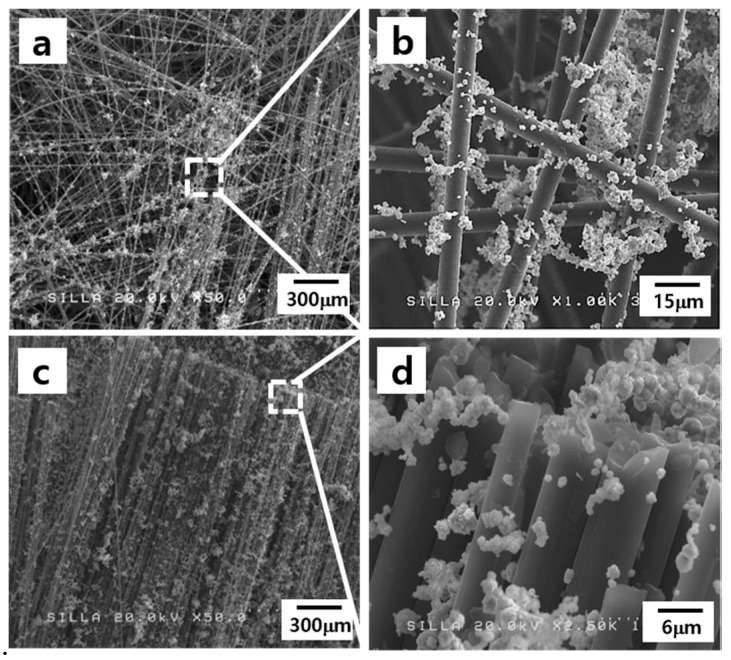
FESEM images of (**a**) the Ni catalyst powder spread on the nonwoven fabric; (**b**) the magnified image of (**a**); (**c**) the Ni catalyst powder spread on the woven fabric; and (**d**) the magnified image of (**c**).

After the Ni catalyst powder was spread on the surfaces of the nonwoven and woven fabrics using a paintbrush, relatively well-dispersed Ni catalyst particles could be observed on the surfaces of the nonwoven and woven fabrics, as shown in Figure 6. Most of these Ni catalyst particles appeared to be located on the surfaces of the constituent CFs (Figure 6b,d).

**Figure 7:**
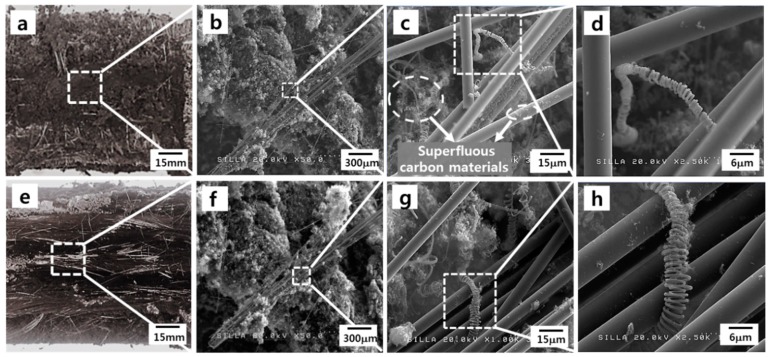
The CF-CMC hybridized nonwoven fabric showing (**a**) optical photograph; (**b**) the FESEM image of (**a**); and (**c**) the magnified-FESEM image of (**b**); and (**d**) the magnified-FESEM image of (**c**) and the CF-CMC hybridized woven fabric showing (**e**) optical photograph; (**f**) the FESEM image of (**e**); (**g**) the magnified-FESEM image of (**f**) and (**h**) the magnified-FESEM image of (**g**).

After the CF-CMC hybrid formation reaction using the TCVD system, the surface morphologies of the CF-CMC hybrids in the nonwoven and woven fabrics were examined, and the imaging results are shown in Figure 7. In both cases, the CF-CMC hybrids were successfully formed. The diameters and the coil pitch dimensions of CMC in the woven fabric seemed to be larger than those in the nonwoven fabric (Figure 7d vs. Figure 7h).

**Figure 8:**
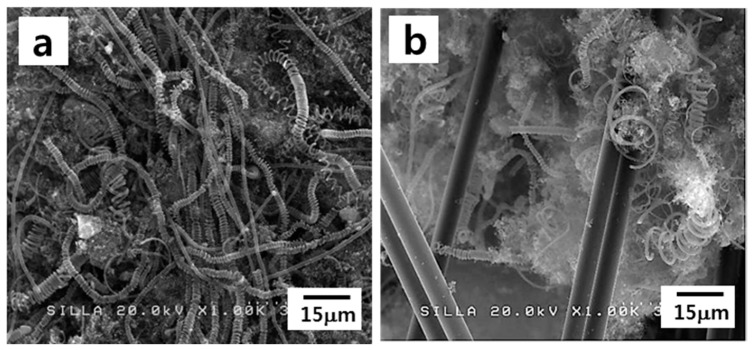
FESEM images showing the locations of the highest density of formed CMCs (**a**) in the nonwoven fabric and (**b**) in the woven fabric cases.

Previously, we reported the growth mode of CMCs, as follows [16]: The formation of CMCs starts owing to the different precipitating rates of two carbon nanofilaments. Thus, the balance axis of the catalyst collapses, giving rise to the spinning of the catalyst and creating the double helix-type geometry. Based on our growth mode of CMCs, the diameters and the coil pitch dimensions of CMCs were understood to be reduced with the development of the double helix geometry. Therefore, the observed smaller diameters and coil pitch dimensions of the CMCs in the nonwoven fabric suggest that the formation of the CMCs progresses further in the case of the nonwoven fabric compared with the woven fabric. Indeed, the density of the formed CMCs in the nonwoven fabric appeared to be much higher than that in the woven fabric (Figure 8a vs. Figure 8b).

The resistivity values of the nonwoven and woven fabrics and their hybrids were estimated using a four-point probe as shown in Table 2. Due to the hybrid formation between CFs and CMCs and the considerable thickness of the samples, the measured values by a four-point probe in this work could not reveal the exact electrical conductive values of the samples. So, we merely compared the electrical conductive characteristics between the native fabrics and the hybridized-fabrics using these estimated values, and then examined the variation of the electrical conductive characteristics of the samples by the hybrid formation as follows.

In the case of the nonwoven fabric, the resistivity seems to be slightly increased by the CF-CMC hybrid formation. This increase appeared to be owing to the formation of superfluous carbon materials in the inter-CF space or on the surfaces of individual CFs in the nonwoven fabric, as shown in Figure 7c.

**Figure 9:**
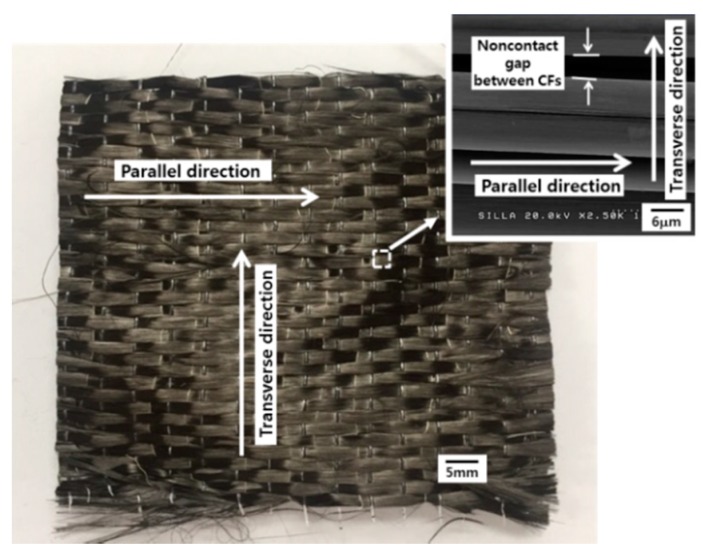
Woven-fabric, showing the parallel and transverse directions for measuring the sheet resistance.

**Figure 10:**
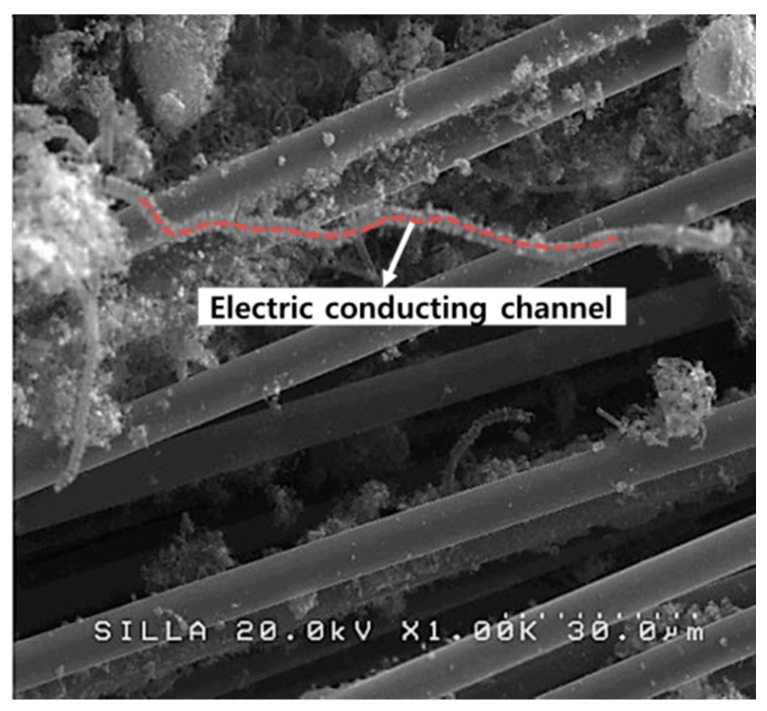
FESEM image, showing the interconnection between CFs by CMCs in the case of the woven fabric.

In the case of the woven fabric, the variation of the resistivity induced by the CF-CMC hybrid formation reaction depended on the measuring direction. The resistivity for the transverse and parallel directions to the individual CFs was shown to be much different as shown in Table 2. This remarkable difference appeared to be owing to the characteristics of the woven fabric. Namely, the direction transverse to individual CFs features noncontact gaps between CFs (Figure 9, inset), which increases the fabric’s resistivity. After the CF-CMC hybrid formation reaction, the resistivity in the direction transverse to individual CFs decreased. The mechanism underlying this electrical conductivity improvement appeared to be the formation of inter-contacts by CMCs in the noncontact gaps between CFs. As in the previous report, the electron-conducting channels in the direction transverse to individual CFs appeared to form by the formation of CMCs among CFs (Figure 10) [17]. Therefore, the created electron-conducting channels may increase the electron conductivity in the direction transverse to individual CFs. For the direction parallel to individual CFs in the woven fabric, the resistivity seemed to be slightly increased. The direction parallel to individual CFs already features continuous connecting CFs. After the CF-CMC hybrid formation reaction, therefore, the electrical conductivity in the direction parallel to individual CFs appeared to be reduced, owing to the formation of superfluous carbon materials in the woven fabric, similar to the nonwoven case.

**Figure 11:**
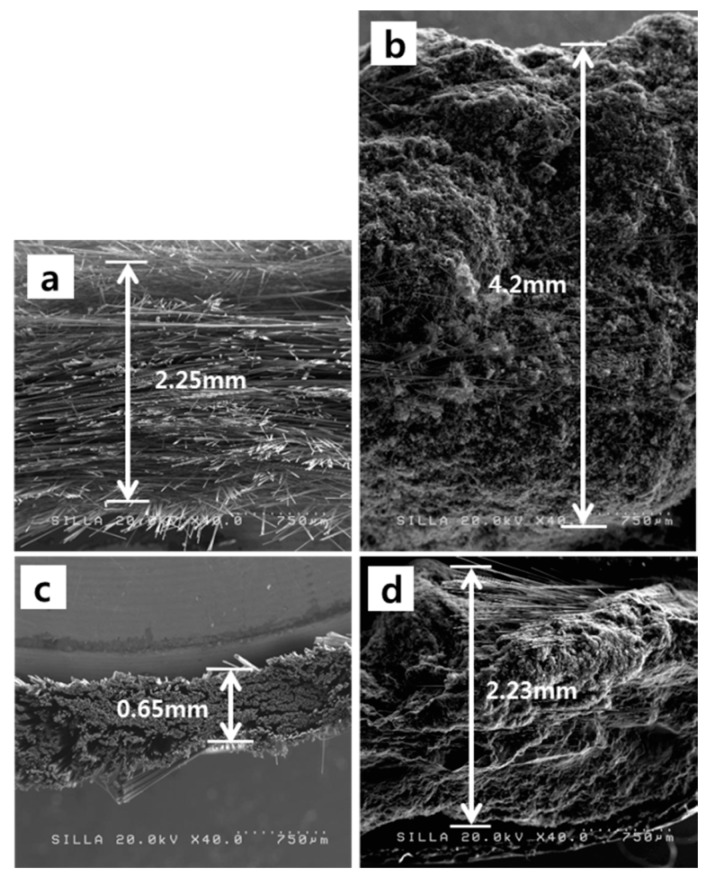
FESEM image, showing the cross-sections of (**a**) the nonwoven fabric; (**b**) the nonwoven fabric after the CF-CMC hybrid reaction; (**c**) the woven fabric; and (**d**) the woven fabric after the CF-CMC hybrid reaction.

Following the hybrid formation between CFs and the superfluous carbon material formation in the fabrics, the thicknesses of the CF-CMC hybridized fabrics increased (Figure 11a,b). Compared with the native fabrics, the thicknesses of the CF-CMC hybridized fabrics increased ~1.87-fold for the nonwoven fabric and ~3.43-fold for the woven fabric (Figure 11).

**Figure 12:**
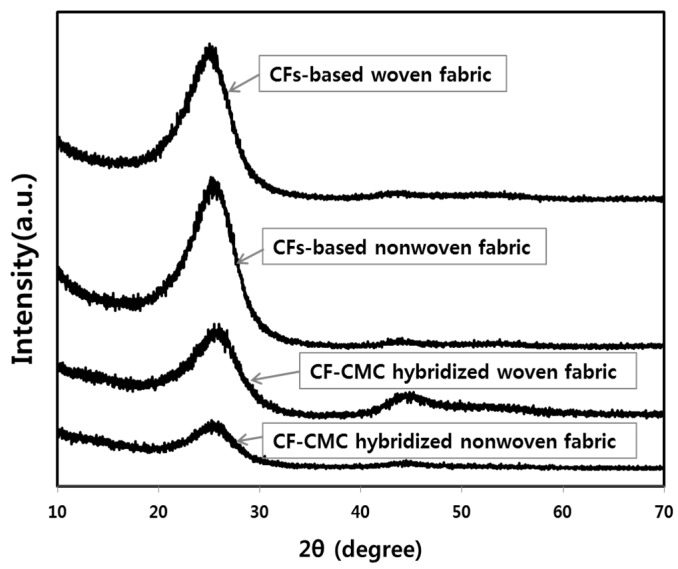
X-ray diffraction (XRD) spectra of the CF-based nonwoven and woven fabrics, and the spectra of their corresponding CF-CMC hybrids.

The crystal structures of the nonwoven and woven fabrics and their corresponding hybrids were investigated using XRD. Figure 12 reveals two peaks, at 24.0° and 43.0°, associated with the typical graphitic (002) and (100)/(101) planes, respectively [18]. Comparing the (002) peaks of the different samples, it is noticed that the intensities for the native fabrics are higher than those for their corresponding hybrids. This suggests that the hybridization reaction of the fabrics likely deteriorated the crystal structure of the CF-based fabrics. Similar to the case of electrical conductivity, the formation of superfluous carbon materials in the fabrics may be regarded as the main reason for the deterioration of the crystal structure of the CF-CMC hybridized fabrics.

**Figure 13:**
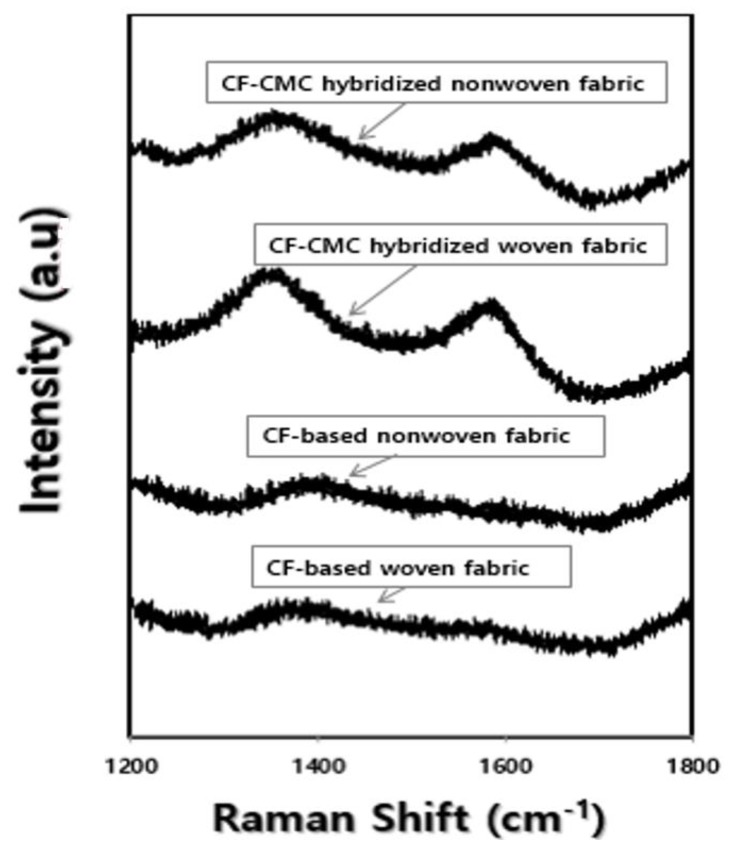
Raman spectra of the CF-based nonwoven and woven fabrics, and the spectra of their corresponding CF-CMC hybrids.

The carbon microcoil quality of the CF-CMC hybridized nonwoven and woven fabrics was investigated using Raman spectroscopy, and the results are shown in Figure 13. The *D* and *G* peaks in the samples were observed around 1350 cm^−1^ and 1600 cm^−1^, respectively [17]. From the curve fitting of the Raman spectra, the corresponding *I_D_*/*I_G_* values were calculated [19]. Table 3 shows the *I_D_*/*I_G_* values and the peak top positions of the *D* and *G* bands, for the CF-CMC hybridized nonwoven and woven fabrics. The *D* and *G* bands are known to be associated with the disordered states of *sp*^2^-hybridized carbon and the stretching vibrations of graphite, respectively [20,21]. As shown in Table 3, the *I_D_*/*I_G_* value for the CF-CMC hybridized nonwoven fabric was lower than that for the CF-CMC hybridized woven fabric, indicating the stronger presence of the ordered carbon phase. In addition, the peak top positions of the *G* and *D* bands for the CF-CMC hybridized nonwoven fabric shifted downward and upward, respectively. These trends also indicate the more pronounced polycrystalline structure associated with well-developed carbon microcoils, in the case of the CF-CMC hybridized nonwoven fabric. Based on these results, we confirmed that the nonwoven fabric, compared with the woven fabric, promotes the formation of carbon microcoils with a regular crystal structure.

To investigate the enhancement of the SE of the CF-based nonwoven and woven fabrics owing to the formation of CF-CMC hybrids, the SE values for the nonwoven fabric, CF-CMC hybridized nonwoven fabric, woven fabric, and CF-CMC hybridized woven fabric were measured in the X-band region (8.0–12.0 GHz). Above all, the observed SE values of the nonwoven fabrics in this work, regardless of the CF-CMC hybrid formation, were above 50 dB throughout the entire range of operating frequencies. Compared with the previously reported SE values, the presently measured values rank higher among the reported SE values (Table 4). Based on this, we suggest that nonwoven fabrics can be effectively used in diverse industrial fields.

**Figure 14:**
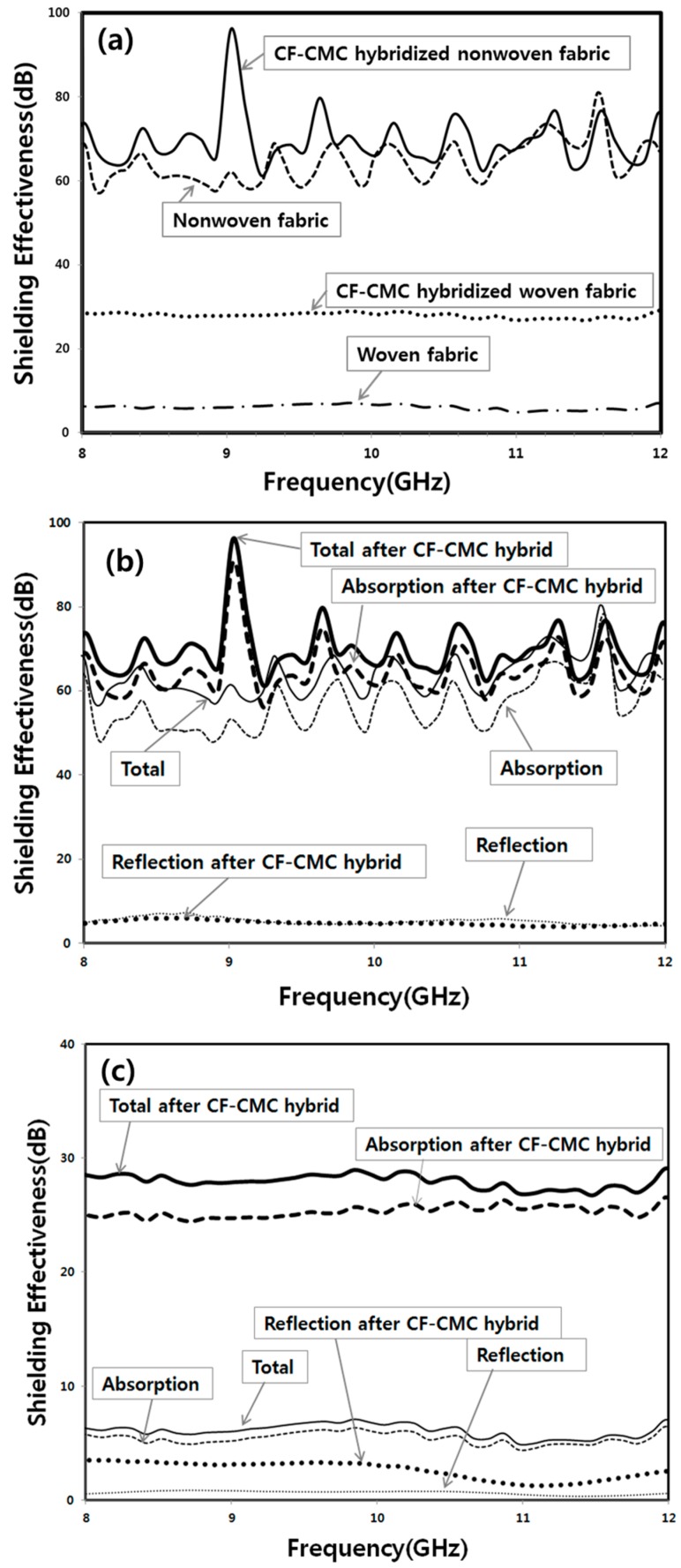
(**a**) Overall SE of the CF-based fabric and its corresponding CF-CMC hybrid, (**b**) overall SE, absorption of SE, and reflection of SE for the nonwoven fabric and its CF-CMC hybrid, and (**c**) overall SE, absorption of SE, and reflection of SE for the woven fabric and its corresponding CF-CMC hybrid.

Figure 14a shows that the SE values of the nonwoven fabric are generally much higher than those of the woven fabric, irrespective of the CF-CMC hybrid formation. The skin depth (δ) of a shield is defined as δ = (πσ*f*μ)^−1/2^ [2]. Namely, δ^2^ is inversely proportional to the electrical conductivity (σ), frequency (*f*), and the magnetic permeability (μ). Higher electrical conductivity can efficiently reduce the skin depth of a shield. In addition, the SE of the EM interference for electrically conducting materials may be estimated using the empirical equation that was introduced by Simon [32], SE = 50 + 10log_10_(ρ*f*)^−1^ + 1.7*t*(*f*/ρ)^1/2^. In this equation, SE is reported in dB, ρ is the resistivity (Ω cm) at room temperature, *t* is the thickness of the sample (cm), and *f* is the operating frequency, respectively. This equation also suggests that increasing the electrical conductivity increases the SE value. Therefore, the higher electrical conductivity value of the nonwoven fabric, compared with that for the direction transverse to individual CFs in the woven fabric, could be attributed to the improved SE values (Table 4). Furthermore, many CFs in the nonwoven fabric crossed with each other. These cross-points may contribute to multiple reflections in the fabric, helping to shield EM waves. Consequently, the SE of the nonwoven fabric will be significantly improved, as in the case of nanoscale fillers in a composite.

As shown in Figure 14b, the SE values for the nonwoven fabric slightly increased following the CF-CMC hybrid formation, although the electrical conductivity of the nonwoven fabric was slightly lowered by the CF-CMC hybridization reaction (Table 2, electrical conductivity of the nonwoven fabric ((2.40 ± 0.23) × 10^3^ S/m) vs. that of the CF-CMC hybridized nonwoven fabric ((1.48 ± 0.77) × 10^2^ S/m). This discrepancy between the improvement of the SE values and deterioration of the electrical conductivity can be explained as follows. The thickness of the nonwoven fabric significantly increases following the CF-CMC hybrid formation (Table 2, thickness of the nonwoven fabric (2.12 ± 0.22 mm) vs. that of the CF-CMC hybridized nonwoven fabric (4.35 ± 1.09 mm)). Thus, a thicker CF-CMC hybridized nonwoven fabric is expected to have more multiple reflection points. The empirical equation of Simon, namely SE ∝ 1.7*t*(*f*/ρ)^1/2^, also reveals that the SE increases owing mostly to the absorption mechanism, as the thickness (t) of the shielding material increases. Furthermore, the intrinsic characteristics of CMCs in the CF-CMC hybridized nonwoven fabric, namely the ability to generate a magnetic field and consequently absorb the incoming EM wave, can facilitate the absorption loss of EM waves at high operating frequencies. Therefore, the SE value for the nonwoven fabric increases following the CF-CMC hybrid formation in spite of the deterioration of its electrical conductivity.

As shown in Figure 14c, the increase in the SE of the CF-CMC hybridized woven fabric was more than twofold throughout the entire range of operating frequencies. This dramatic increase in the SE value of the CF-CMC hybridized woven fabric seemed to be mainly ascribed to the enhanced electrical conductivity, particularly in the direction transverse to individual CFs (from (0.82 ± 0.14) × 10 S/m to (1.82 ± 0.20) × 10^2^ S/m) following the CF-CMC hybrid formation (Table 2).In addition, as in the case of the nonwoven fabric, the thickness of the woven fabric was significantly increased by the CF-CMC hybrid formation (Table 2, thickness of the woven fabric (0.56 ± 0.04 mm) vs. that of the CF-CMC hybridized woven fabric (2.77 ± 0.34 mm). Furthermore, the intrinsic characteristics of CMCs in the CF-CMC hybridized woven fabric can enhance the absorption loss of EM waves at high operating frequencies. Consequently, the SE values of the CF-CMC hybridized woven fabric can be significantly improved by the SE absorption mechanism, owing to the increase in its thickness and electrical conductivity and owing to the intrinsic characteristics of CMCs.

## 4. Conclusions

In this work, the SE of the nonwoven fabric was much higher than that of the woven fabric, irrespective of the CF-CMC hybrid formation. The higher electrical conductivity of the nonwoven fabric compared with that of the woven fabric in the direction transverse to individual CFs, as well as many CF cross-points in the nonwoven fabric, seem to be the main reason for the observed increase in the SE value. Despite the deterioration of the electrical conductivity, the SE of the CF-CMC hybridized nonwoven fabric seems to be slightly increased owing to the increased thickness and the intrinsic characteristics of CMCs. Owing to the increased thickness and increased electrical conductivity, the SE value of the CF-CMC hybridized woven fabric was nearly twofold higher than that of the native woven fabric, throughout the entire range of operating frequencies. Finally, the SE of the presently studied CF-CMC hybridized nonwoven fabric was above 60 dB throughout the entire range of operating frequencies, from 8 to 12 GHz. Consequently, the CF-CMC hybridized nonwoven fabric is promising for use in diverse applications that require EM wave shielding. In industrial points of view, the production cost and the mass production are important. For the native carbon-based nonwoven fabric, the production cost is about 50 $/kg. So, the native carbon-based nonwoven fabric itself seems to be efficient for the application in the diverse industrial fields. In the near future, we are sure that the production cost-reduced hybridized nonwoven fabric would be applicable even for the high value-added industry.

## Figures and Tables

**Table 1:** Experimental conditions for the formation of the carbon fiber-carbon microcoil (CF-CMC) hybrid materials.

C_2_H_2_ Flow Rate (sccm)	CS_2_ Flow Rate (sccm)	Total Pressure (torr)	Total Reaction Time (min)	Substrate Temp. (°C)
500	15	100	60	750

**Table 2:** Thickness, volume resistivity, and electrical conductivity of the nonwoven and woven fabrics and their hybridized fabrics.

Samples and Measuring Direction		Thickness t (mm)	Volume Resistivity ρ (Ω∙cm)	Electrical Conductivity σ (S/m)	*Correction Factor F (w/s)
**Nonwoven fabric**		2.12 ± 0.22	(4.10 ± 0.11) × 10^−2^	(2.44 ± 0.06) × 10^3^	~0.98
**CF-CMC hybridized nonwoven fabric**		4.35 ± 0.11	(7.18 ± 0.17) × 10^−1^	(1.39 ± 0.03) × 10^2^	~0.78
**Woven fabric**	**Direction transverse to individual CFs**	0.56 ± 0.04	(1.23 ± 0.22) × 10	8.34 ± 1.50	~0.99
	**Direction parallel to individual CFs**		3.07 ± 0.01	(3.26 ± 0.02) × 10	~0.99
**CF-CMC hybridized woven fabric**	**Direction transverse to individual CFs**	2.77 ± 0.34	(5.12 ± 0.62) × 10^−1^	(1.98 ± 0.24) × 10^2^	~0.93
	**Direction parallel to individual CFs**		(4.46 ± 0.54) × 10^−1^	(2.27 ± 0.27) × 10^2^	~0.93

* Correction factor was calculated from Table 3 of Smits work [15].

**Table 3:** Peak locations for the *G* and *D* bands in the Raman spectra, and *I_D_*/*I_G_* ratios.

Samples and Measuring Direction	*G*-Band Peak-Top (cm^−1^)	*D*-Band Peak-Top (cm^−1^)	*I_D_*/*I_G_*
After CF-CMC hybrid formation reaction of nonwoven fabric	1586.6	1356.2	0.9922
After CF-CMC hybrid formation reaction of woven fabric	1594.0	1337.0	1.0071

**Table 4:** Previously reported shielding effectiveness values of carbon-based materials.

Various Carbon-Based Materials	Density (g/cm^3^)	Thickness (mm)	Conductivity (S/m)	Operating Frequency (GHz)	EMI SE (dB)	Refs.
*PMMA/graphene	0.79	2.40	3.11	8–12	13–19	[22]
*PS/graphene	0.27	2.50	2.20 × 10^−1^	8.2–12.4	17–22	[23]
*PDMS/graphene	0.06	1.00	2.00 × 10^2^	8–12	18–40	[9]
*PEI/graphene	0.29	2.30	2.20 × 10^−5^		18–23	[24]
*PI/graphene	0.28	0.80	8.00 × 10^−1^		17–21	[25]
Graphene-foam	0.06	0.30	3.10 × 10^2^	8.2–12.5	23–27	[26]
Multilayer-graphene paper	1.09	0.50 × 10^−1^	1.40 × 10^5^	8.2–12.4	62.5–74.8	[27]
Iodine-doped *LG paper	–	0.12 × 10^−1^	1.05 × 10^5^	8.2–12.5	50–55	[28]
*CNF-GN	0.08–0.10	0.22–0.27	8.00 × 10^2^	8.2−12.4	26-28	[7]
Graphene aerogel/carbon texture	0.07	3.00	–	8–12.5	36–37	[29]
Fluorocarbon polymer/MWCNT	1.70	3.80	–	8.2–12.4	46–52	[30]
Fluorocarbon polymer/MWCNT foam	1.20	3.80	–		42–45	
Nickel, Silver (Ni/Ag) plated nylon fabric.	–	0.10	3.33 × 10^2^	0.03–10	60	[31]
Nickel, Copper, Silver (Ni/Cu/Ag) plated Nylon fabric	–	0.12	8.89 × 10^3^		95	
Nonwoven fabric	0.06	2.12 ± 0.22	(2.40 ± 0.23) × 10^3^	8–12	58–70	*This work*
CF-CMC hybridized nonwoven fabric	0.29	4.35 ± 0.11	(1.39 ± 0.03) × 10^2^		62–98	
Woven fabric	0.44	0.56 ± 0.04	Transverse: (0.82 ± 0.14) × 10^1^		5–8	
			Parallel: (3.23 ± 0.02) × 10^1^			
CF-CMC hybridized woven fabric	0.59	2.77 ± 0.34	Transverse: (1.82 ± 0.20) × 10^2^		27–30	
			Parallel: (2.11 ± 0.25) × 10^2^			

*PMMA: polymethylmethacrylate, *PS: polystyrene, *PDMS: poly(dimethyl siloxane), *PEI: polyetherimide, *PI: polyimide, *LG: large-sized graphene sheets, *CNF-GN: carbon nanofiber-graphene nanosheet.

## References

[1] Wu J., Chung D.L. (2002). Increasing the Electromagnetic Interference Shielding Effectiveness of Carbon Fiber Polymer-matrix Composite by Using Activated Carbon Fibers. Carbon.

[2] Chung D.L. (2001). Electromagnetic Interference Shielding Effectiveness of Carbon Materials. Carbon.

[3] Yang S., Lozano K., Lomeli A., Foltz H.D., Jones R. (2005). Electromagnetic Interference Shielding Effectiveness of Carbon Nanofiber/LCP Composites. Compos. Part A-Appl. Sci. Manuf..

[4] Sau K.P., Chaki T.K., Chakraborty A., Khastgir D. (1997). Electromagnetic Interference Shielding by Carbon Black and Carbon Fiber Filled Rubber Composites. Plast. Rubber Compos. Process. Appl..

[5] Motojima S., Hoshiya S., Hishikawa Y. (2003). Electromagnetic Wave Absorption Properties of Carbon Microcoils/PMMA Composite Beads in W Bands. Carbon.

[6] Zhao D.L., Shen Z.M. (2008). Preparation and Microwave Absorption Properties of Carbon Nanocoils. Mater. Lett..

[7] Song W.L., Wang J., Fan L.Z., Li Y., Wang C.Y., Cao M.S. (2014). Interfacial Engineering of Carbon Nanofiber−Graphene−Carbon Nanofiber Heterojunctions in Flexible Lightweight Electromagnetic Shielding Networks. ACS Appl. Mater. Interface.

[8] Song W.L., Fan L.Z., Cao M.S., Lu M.M., Wang C.Y. (2014). Facile Fabrication of Ultrathin Graphene Papers for Effective Electromagnetic Shielding. J. Mater. Chem. C.

[9] Chen Z., Xu C., Ma C., Ren W., Cheng H.M. (2013). Lightweight and Flexible Graphene Foam Composites for High-Performance Electromagnetic Interference Shielding. Adv. Mater..

[10] Kang G.-H., Kim S.-H. (2016). Effect of Incorporating Carbon Nanocoils on the Efficiency of Electromagnetic-Wave Shielding of Carbon-Nanomaterial Composites. Appl. Surf. Sci..

[11] Kang G.-H., Kim S.-H., Park S. (2017). Enhancement of Shielding Effectiveness for Electromagnetic Wave Radiation Using Carbon Nanocoil-Carbon Microcoil Hybrid Materials. Appl. Surf. Sci..

[12] Liu L., Zhou K., He P., Chen T. (2013). Synthesis and microwave absorption properties of carbon coil–carbon fiber hybrid materials. Mater. Lett..

[13] Liu L., He P., Zhou K., Chen T. (2014). Microwave Absorption Properties of Carbon Fibers with Carbon Coils of Different Morphologies (Double Microcoils and Single Nanocoils) Grown on Them. J. Mater. Sci..

[14] Lee N.-Y., Kim S.-H. (2018). Enhanced Formation of the Carbon Microcoils by the Stepwise Type Manipulation of CS_2_ Flow Injection. Key Eng. Mater..

[15] Smits F. (1958). Measurement of Sheet Resistivities with the Four-Point Probe. Bell Syst. Tech. J..

[16] Park S., Jeon Y.-C., Kim S.-H. (2013). Effect of Injection Stage of SF_6_Flow on Carbon Micro Coils Formation. ECS J. Solid State Sci. Technol..

[17] Sevilla M., Fuertes A.B. (2009). Easy Synthesis of Graphitic Carbon Nanocoils from Saccharides. Mater. Chem. Phys..

[18] Bi H., Kou K.C., Ostrikov K., Yan L.K., Zhang J.Q., Ji T.Z., Wang Z.C. (2009). Unconventional Ni–P Alloy-Catalyzed CVD of Carbon Coil-like Micro- and Nano-Structures. Mater. Chem. Phys..

[19] Yang S., Chen X., Katsuno T., Motojima S. (2007). Controllable Synthesis of Carbon Microcoils/Nanocoils by Catalysts Supported on Ceramics Using Catalyzed Chemical Vapor Deposition Process. Mater. Res. Bull..

[20] Robertson J. (2002). Diamond-like Amorphous Carbon. Mater. Sci. Eng..

[21] Sajitha E.P., Prasad V., Subramanyam S.V., Eto S., Takai K., Enoki T. (2004). Synthesis and Characteristics of Iron Nanoparticles in a Carbon Matrix along with the Catalytic Graphitization of Amorphous Carbon. Carbon.

[22] Zhang H.B., Yan Q., Zheng W.G., He Z., Yu Z.Z. (2011). Tough Graphene-polymer Microcellular Foams for Electromagnetic Interference Shielding. ACS Appl. Mater. Inter..

[23] Yan X., Ren P.G., Pang H., Fu Q., Yang M.B., Li Z.M. (2012). Efficient Electromagnetic Interference Shielding of Light Weight Graphene/Polystyrene Composite. J. Mater. Chem..

[24] Ling J.Q., Zhai W.T., Feng W.W., Shen B., Zhang J.F., Zheng W.G. (2013). Facile Preparation of Lightweight Microcellular Polyetherimide/Graphene Composite Foams for Electromagnetic Interference Shielding. ACS Appl. Mater. Inter..

[25] Li Y., Pei X.L., Shen B., Zhai W.T., Zhang L.H., Zheng W.G. (2015). Polyimide/Graphene Composite Foam Sheets with Ultrahigh Thermostability for Electromagnetic Interference Shielding. RSC Adv..

[26] Shen B., Li Y. (2016). Microcellular Graphene Foam for Improved Broadband Electromagnetic Interference Shielding. Carbon.

[27] Paliotta L., Sarto M.S. (2015). Highly Conductive Multilayer-Graphene Paper as a Flexible Lightweight Electromagnetic Shield. Carbon.

[28] Wan Y.-J. (2017). Graphene Paper for Exceptional EMI Shielding Performance Using Large-Sized Graphene Oxide Sheets and Doping Strategy. Carbon.

[29] Song W.-L., Guan X.T., Fan L.-Z., Cao W.Q., Wang C.Y., Cao M.S. (2015). Tuning Three Dimensional Textures with Graphene Aerogels for Ultra-Light Flexible Graphene/Texture Composites of Effective Electromagnetic Shielding. Carbon.

[30] Fletcher A., Gupta M.C., Dudley K.L., Vedeler E. (2010). Elastomer Foam Nanocomposites for Electromagnetic Dissipation and Shielding Applications. Compos. Sci. Technol..

[31] Statex, Shieldex US. https://www.shieldextrading.net/products/fabrics/.

[32] Simon R.M. (1981). EMI Shielding Through Conductive Plastics. Polym. Plast. Technol. Eng..

